# Magnetic Resonance Imaging Compatible Non-Invasive Fibre-Optic Sensors Based on the Bragg Gratings and Interferometers in the Application of Monitoring Heart and Respiration Rate of the Human Body: A Comparative Study

**DOI:** 10.3390/s18113713

**Published:** 2018-10-31

**Authors:** Jan Nedoma, Stanislav Kepak, Marcel Fajkus, Jakub Cubik, Petr Siska, Radek Martinek, Petr Krupa

**Affiliations:** 1 Department of Telecommunications, Faculty of Electrical Engineering and Computer Science, VSB-Technical University of Ostrava, 17 Listopadu 15, 70833 Ostrava, Czech Republic; stanislav.kepak@vsb.cz (S.K.); marcel.fajkus@vsb.cz (M.F.); jakub.cubik@vsb.cz (J.C.); petr.siska@vsb.cz (P.S.); 2 Department of Cybernetics and Biomedical Engineering, Faculty of Electrical Engineering and Computer Science, VSB-Technical University of Ostrava, 17 Listopadu 15, 70833 Ostrava, Czech Republic; radek.martinek@vsb.cz; 3 Department of Imaging Method, Faculty of Medicine, University of Ostrava, Syllabova 19, 70300 Ostrava, Czech Republic; petr.krupa@osu.cz

**Keywords:** interferometer, Bragg grating, heart rate (HR), respiratory rate (RR), phonocardiography (PCG), ballistocardiography (BCG), electrocardiography (ECG), polydimethylsiloxane (PDMS), vital signs, non-invasive measurements, patient monitoring, biomedical engineering, magnetic resonance imaging (MRI)

## Abstract

The publication presents a comparative study of two fibre-optic sensors in the application of heart rate (HR) and respiratory rate (RR) monitoring of the human body. After consultation with clinical practitioners, two types of non-invasive measuring and analysis systems based on fibre Bragg grating (FBG) and fibre-optic interferometer (FOI) have been designed and assembled. These systems use probes (both patent pending) that have been encapsulated in the bio-compatible polydimethylsiloxane (PMDS). The main advantage of PDMS is that it is electrically non-conductive and, as well as optical fibres, has low permeability. The initial verification measurement of the system designed was performed on four subjects in a harsh magnetic resonance (MR) environment under the supervision of a senior radiology assistant. A follow-up comparative study was conducted, upon a consent of twenty volunteers, in a laboratory environment with a minimum motion load and discussed with a head doctor of the Radiodiagnostic Institute. The goal of the laboratory study was to perform measurements that would simulate as closely as possible the environment of harsh MR or the environment of long-term health care facilities, hospitals and clinics. Conventional HR and RR measurement systems based on ECG measurements and changes in the thoracic circumference were used as references. The data acquired was compared by the objective Bland–Altman (B–A) method and discussed with practitioners. The results obtained confirmed the functionality of the designed probes, both in the case of RR and HR measurements (for both types of B–A, more than 95% of the values lie within the ±1.96 SD range), while demonstrating higher accuracy of the interferometric probe (in case of the RR determination, 95.66% for the FOI probe and 95.53% for the FBG probe, in case of the HR determination, 96.22% for the FOI probe and 95.23% for the FBG probe).

## 1. Introduction

This publication builds on current trends in non-invasive monitoring of vital functions [1]. The original benefit of this work is the comparison of two types of fibre-optic systems, namely the fibre-optic interferometric (FOI) system and the fibre Bragg grating (FBG) system, and their verification against conventional clinical systems in use. The electrocardiograph (ECG) and the contact elastic band with the built-in Piezo-Electric Respiration Transducer (PERT) were used as a reference. The comparative study is focused on human body pulse rate (HR) and respiratory rate (RR) monitoring for the harsh magnetic resonance (MR) environment (e.g., Magnetic Resonance, X-ray, UV absorption and radiation, etc.). Advantageous characteristic features of fibre-optic sensors include their independence from an active power supply and a high immunity to electromagnetic interference. Thanks to these attributes, these sensors can be used with other electronic equipment without generating electrical noise that may compromise the quality of vital sign monitoring and potentially lead to patient safety concerns. Fibre-optic sensors are gaining more popularity due to their flexibility, very small dimensions, and reliability. Please see the articles below.

In the research part of this study, two non-invasive probes based on FBG (hereinafter the FBG probe) and interferometer (hereinafter the FOI probe) were designed and assembled. These probes are encapsulated in the polydimethylsiloxane (PMDS) polymer, which offers a unique combination of suitable properties for biomedical applications. The main advantage of PDMS is that it is inert to human skin, it is electrically non-conductive, it is a diamagnetic substance and it is resistant to UV radiation [2]. Other benefits include its mechanical and heat resistance, washability and repeatability. The results presented in [3,4,5,6,7] indicate that these types of encapsulation do not affect the structure and function of the FBG and FOI probes.

FBG and FOI probes have been designed in consultation with clinical practitioners and devised in such a way that they were as small as possible and most suitable for application in the field of biosignal measurement. While the FBG probe can be constructed as extremely compact, the FOI sensor contains short sections of optical fibre, where the limiting factor is the critical bend radius of the fibre, which is set to 3 cm for the most common G.652.D single-mode fibres [8]. The FOI sensor uses connection of the Mach–Zehnder interferometer by means of a coupler with three output ports. The measuring FOI and FBG probes are part of a contact elastic band located on the patient’s chest (in this study, this elastic band is replaced with an elastic band with a built-in PERT). Measurement with the FBG probe is based on sensing the movement of the patient’s thoracic cavity; in the case of the FOI probe, which is more sensitive, it is also possible to record the murmurs and sounds emitted during the cardiac cycle. Along with this, expansion of the fibre-optic sensors due to the respiratory activity is also measured.

Table 1 shows a comparison of both systems and takes into account their advantages/disadvantages/applications possibilities and price of systems/probes.

The main assumed and current area from the point of view of clinical practice is the possibility to apply such sensors for patients’ HR and RR monitoring in the harsh MR environment, or for the prediction of the so-called hyperventilation states that occur during these examinations due to e.g., a closed tunnel environment [9,10]. These are cases when the person begins to breathe faster and their heart rate increases (tachycardia). Spontaneous breathing is constantly at risk due to anaesthetics, upper airway obstruction, or panic and claustrophobic conditions during MR examinations. Another advantage of the fibre-optic probes is the potential to simplify vital sign monitoring and consequently improve the comfort level of patients in long-term health care facilities, hospitals and clinics, as one sensor can be used to monitor RR and HR simultaneously.

In the first phase of the comparative study, the fibre Bragg gratings and FOI systems and probes were tested in a harsh MR environment under the supervision of a senior radiology assistant, their properties were then verified by long-term testing in a laboratory environment and discussed with the head radiologist doctor (Petr Krupa). The real issue is to obtain consent from the ethics committee to carry out a larger study on a larger number of test subjects within the MR environment or in the environment of the long-term health care facilities, hospitals and clinics. For this reason, the article is drawn up in such a way that, after testing the basic functionality of the measuring systems and probes in the MR environment during standard examinations on four test subjects and within a limited time interval of tens of minutes, see Section 4.1, the study was moved to a laboratory environment, where it could be performed on a larger sample of test subjects (different age, weights, heights, and sexes) at long time intervals (tens of hours) against the HR and RR reference commonly used in clinical practice (see Section 4.2).

On the basis of the literary research below, it can be concluded that a similar comparative study of such an extent has not been carried out and that further use of the proposed measurement systems and probes in clinical practice was necessary. The article documents the accuracy of the probes tested compared to the HR and RR references. The results of this study now serve as a stimulus to get approval from the Ethics Committee for Clinical Trials; a major clinical study in MR environment and in long-term health care facilities, hospitals and clinics is being planned.

## 2. State of the Art

HR measurement is an important factor that can provide information about changes in the blood circulation and heart activity; therefore, it is often a measured quantity in the medical environment. Today, various quantities, such as the ECG electrical signal, the acoustic signal, blood pressure changes in the circulatory system, changes in tissue volume as a result of volume changes in the circulatory system, changes in tissue impedance associated with changes in the blood volume in a specific tissue section or changes in blood flow velocity—due to changes in blood pressure in the circulatory system—are used for its determination [11].

Measurement by means of an electric ECG signal is nowadays a generally recognized standard [12,13,14,15] although this method of determining HR has existed for decades. To measure the twelve lead ECGs, a connection of three limb electrodes (both upper limbs and left lower limb), one ground electrode (right lower limb) and six chest electrodes are used. A one-lead ECG using three electrodes can also be used to determine the HR, which has been dealt within the study by the authors [12].

Determining HR by means of an acoustic signal (phonocardiography) usually uses sensitive microphones that are located in the chest area and its surroundings. The algorithms are then applied to the resulting signal as we see in the article [16].

The blood pressure varies during the heart activity and fluctuates between two values (HR). It is the upper limit, systolic pressure—the maximum arterial pressure during the systole, and lower limit, diastolic pressure—the lowest arterial pressure during the diastole. During life, the systolic pressure increases, while the diastolic pressure is a measure of peripheral resistance. Depending on the position of the pressure sensor, we divide pressure measurement methods into direct and indirect [17].

Plethysmography, where body part volume depends on the immediate blood pressure value, is used to evaluate the HR from tissue volume change. This is enabled by the elasticity of the bloodstream as well as the tissues. To do this, a photoelectric plethysmograph, which works either with a translucent sensor or a reflective sensor, is used. Changes in blood pressure alter the volume of the capillaries, which causes a change in absorption, reflection and scattering of light. This method is used to measure volume changes and the heart rate. This can be measured in a wearable device, as shown in the article [18].

The time dependence of tissue impedance changes is an HR function. Its shape is similar to the shape of the time dependence of pressure changes in the bloodstream. In the cardiotachometer, the input part is formed by a reograph, whose output signal is shaped in a suitable shaping circuit. At the output of the shaping circuit, there are pulses, whose frequency corresponds to the heart rate. In this mode of detection, the effect of the stimulator, if the stimulation was not effective, and the extrasystoles, which did not expel the blood from the ventricles into the bloodstream, are automatically excluded from the evaluation. There are a large number of publications dealing with these devices and their modifications [19,20].

The PERTs are commonly used to determine the respiratory rate. They are applied, for example, in sleeping laboratories [21]. These are passive devices not requiring electrical power that generate an electrical signal corresponding to the breathing period. These sensors are fixed to an elastic band that is fastened around the chest. It generates a high-level, linear signal in response to changes in thoracic circumference associated with respiration. These belts, however, cannot be used for quantitative respirative volume measurements. The advantage, however, is that RR belt detection can be very reliable, even with a range of motion artifacts [22], providing a solid reference signal.

One of the most sensitive methods of monitoring vital functions is the use of fibre-optic interferometers. The principle of the function is to measure changes in the electromagnetic wave path due to the action of the forces from the external environment. In the case of interferometers, these changes can be measured with high accuracy at a value level smaller than the wavelength of the light. The sensitivity is so high that a range of measurements can also be performed in indirect contact with the human body. The publication [23] describes a principle of creating a resonator between the body and the radio frequency (RF) aerial to measure the heart and respiratory rates; the resulting signal is very similar to the signal from the photoplethysmogram. The article thus proves that these small changes can also be recorded, even by electromagnetic waves with a much longer wavelength, e.g., 24 GHz [24]. Due to this fact, smart fabrics containing sensors and monitors of wearable electronics [25] are created. It is, therefore, obvious that the principle is functional, even with optical radiation. The basic types of interferometers are multimode ones [26]; they are formed as follows: a part of the measuring optical fibre with other core properties (hollow or no core, a multi-point fibre with a larger core, etc.) is placed between or behind the single-point fibre. The advantage is very cheap production of the sensor, for example based on plastic fibres, described in [27,28].

A sensor assembled on the basis of a speckle interferometer is incorporated in the mattress, thus making the transmission of the mechanical movements between the body and the sensor indirect; linear photodetectors need to be used for scanning. Even so, the results achieved in [27] shown sensitivity and accuracy of heart rate measurement around 98%. Articles [29,30,31,32] were focused on measuring the respiratory rate. Here, a hollow-core fibre sensor was used (photonic crystal fibre). The resulting interference between the core and the cladding mode corresponded to the respiratory rate when the sensor was placed in the respirator mask. The principle is then similar to today’s thermistor respiration sensors that are used in hospital environments. Michelson interferometers have proved more practical; even in this case, the sensor may be in direct or indirect contact with the body, but the respiration and the pulse can be monitored at the same time, as simpler methods and conventional photodetectors can be used for detection [33]. The resulting measurement accuracy of is more than 95%, according to the authors [34,35,36]. Mach–Zehnder interferometers [37], which are characterized by simpler and cheaper production, were also demonstrated. In addition, they can be made using tapers, as shown in [38], dealing with pulse detection.

The second option, described in the article, regarding the method of monitoring vital functions by means of a fibre-optic sensor, is to use an FBG sensor. This type of sensor can be used for monitoring the so-called ballistocardiography (BCG) signals, as the authors of the publications [39,40,41,42,43,44,45,46,47,48,49,50,51,52,53,54,55,56,57,58,59,60,61] have shown. This is a relatively new non-invasive technique that is primarily used to monitor respiratory activity, changes and rhythm of cardiac activity. For example, in the article [39], the authors carried out experimental measurements using an FBG sensor prototype, quantitative and qualitative studies have been performed and the results are found to be reliable and accurate, validating its potential as a standalone medical diagnostic device. An electrical stethoscope was used as the reference signal. The article [43] describes experimental measurements with FBG sensors that can be used to measure the heart rate and the respiratory rate in the sitting position. The analyzed HR and RR results corresponded to the results achieved by commercial devices. Very small relative measurement errors were achieved by the authors of study [56], which focused on the use of the FBG sensor prototype for measuring BCG signals not only in the sitting but also in the standing position of the body. Conventional ECG was used as the reference signal; the FBG sensor prototype was characterized by a relative error of 1.8%. The article [59] describes the use of two FBG sensors encapsulated in the PDMS polymer. The Bland–Altman (B–A) statistical analysis (the study was performed on 1 min recordings) demonstrates the HR detection with a satisfactory accuracy in multiple subjects. For the entire data set, 96.54% of the values lie within the ±1.96 SD (standard deviation) range for the HR determination. Experimental use of FBG sensors directly in the MR environment is described by the studies [41,45] and, for example, by thepublication [46], which provides a solution whereby the FBG sensor is attached to a plexiglas pad on which the patient’s back rests during the examination.

Not many scientific studies or publications dealing with comparison of different signals from different types of sensors have been published. However, no study is based on extensive evaluation of phonocardiographic or ballistocardiographic signals sensed by fibre-optic sensors against conventional ECG devices or respiration sensors. The article [62] describes the initial research focused on comparing synchronous recordings of BCG, ECG, Carotid Pulse Wave, and phonocardiography (PCG) in acute pharmacological trials. The article [63] describes the comparison of the Heart Rate Variability (HRV) indices of the ECG and BCG signals acquired. The results confirmed the feasibility of extracting HR information from all of the BCG experimental series. A partially relevant study to our research is described in the publication [64]. This study includes comparison of three signals, phonocardiography (PCG), electrocardiography (ECG) and photoplethysmography (PPG). However, this article is primarily focused on describing a new approach used to detect the specific parameters and relations between the three biomedical signals used in clinical diagnosis.

Table 2 lists the comparison properties of the most interesting fibre-optic sensors (based on both interferometric and Bragg grating designs) designed for monitoring respiration and heart functions, which were studied and discussed in prior works. The first column provides a reference to the article/s (see the reference sheet). The second column presents the type of interferometer and the physiological parameter(s) that can be monitored. The third column summarizes the most recent experimental studies and their results (if it was published).

## 3. Methods

### 3.1. Interferometric Phonocardiography

The principle of heart rate measurement using the FOI probe is based on the so-called phonocardiography. Mechanical and acoustic activity of the heart and the mechanical functioning of the lungs cause changes in the core refractive index and changes in the length of the measuring optical fibre placed on the body and these microscopic changes in the optical path are appear in the phase delay of ϕ in Equation (1) and its difference Δϕ in Equation (2), where λ is the wavelength of the radiation source, *n* the refractive index of the optical fibre core, *L* is the physical length of the fibre. These changes are then evaluated by the interferometric measuring system:

(1) $$\phi =\frac{2\pi }{\lambda }nL,$$

(2) $$\Delta \phi =\frac{2\pi n}{\lambda }\Delta L+\frac{2\pi }{\lambda }L\Delta n-2\pi L\frac{n}{\lambda^{2}}\Delta \lambda .$$

The activity of the heart is connected with the creation of a large number of characteristic sounds. These sounds occur due to changes in the velocity (or the character) of the blood flow and closing or opening of the respective valves. Hence, this is a diagnostic method that is based on sensing the acoustic signals (heart sounds) described above, which accompany the mechanical vibrations originating in the heart and the blood vessels. The conventional electrocardiogram describes the electrical activity of the heart, while the phonocardiogram describes its mechanical (acoustic) activity.

**Figure 1:**
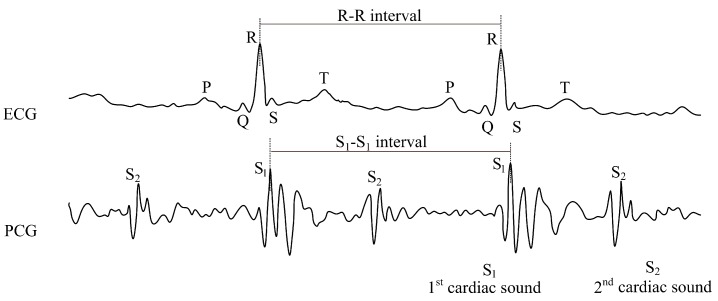
Sample recording of electrocardiogram (ECG) and phonocardiography (PCG) signals.

The phonocardiographic signal consists of two major sound phenomena: the first one, the so-called systolic sounds ($S_{1}$), and, the second one, the so-called diastolic sounds ($S_{2}$) (please see Figure 1). The first sound ($S_{1}$) is associated with the closure of the bicuspid and tricuspid valves at the beginning of the systole, and its commencement corresponds to the ECG R-wave peak. The second sound ($S_{2}$) is induced by the closure of the semilunar valves. The formation and duration of the diastolic sound ($S_{2}$) is associated with the ECG T-wave.

Other two sounds, specifically prodiastolic ($S_{3}$) and presystolic ($S_{4}$), can be detected using the phonocardiogram. The prodiastolic sound is caused by the ventricular muscle vibration in the fast filling phase of the ventricles. The presystolic sound is a manifestation of the ventricular muscle vibration during the chamber systole. These two sounds, however, are not physiological in adults, and their presence is a manifestation of heart insufficiency (the so-called protodiastolic and presystolic gallop) [73,74,75,76]. From the point of view of phonocardiography, frequency, intensity, possible changes or duration of the individual components of the signal are evaluated with the signal being sensed.

### 3.2. Grating Ballistocardiography

The second method of measuring respiratory and heart rates is to use the Bragg grating probe, which consists of a periodic structure of refractive index changes in the optical fibre core. The principle of measuring respiratory and heart rates using the FBG probe is based on the so-called ballistocardiography. The respiratory activity is physiologically manifested by the expansion of the thoracic basket of the human body. This phenomenon exerts pressure on the FBG probe located in the chest area. Due to this pressure effect, the geometrical and optical properties of the FBG probe change, which is reflected by a shift in the spectral domain of reflected light, referred to as the Bragg wavelength:

(3) $$\lambda_{B}=2n_{eff}\Lambda ,$$

where $\lambda_{B}$ is the Bragg wavelength, $n_{eff}$ is the effective refractive index of the grating structure in the fibre and Λ is the spatial period of grating structure refractive index variation. The quantity of the FBG probe spectral response $\Delta \lambda_{B}$ is given by the following relation:

(4) $$\frac{\Delta \lambda_{B}}{\lambda_{B}}=k\varepsilon +\left(\alpha_{\Lambda }+\alpha_{n}\right)\Delta T,$$

where *k* is the deformation coefficient, ε is the acting deformation associated with the pressure exerted by the thoracic basket, $\alpha_{\Lambda }$ is the coefficient of thermal expansion, $\alpha_{n}$ is the thermo-optic coefficient and ΔT is the change in temperature [77].

**Figure 2:**
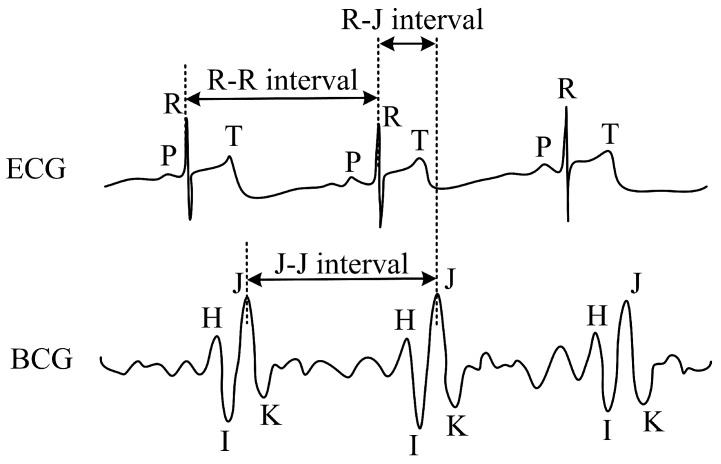
Sample recordings of electrocardiogram (ECG) and ballistocardiography (BCG) signals.

The resulting pressure modulation corresponds to the respiration pattern. The pressure action of the heart activity also occurs in the thoracic area. This response is one to two orders smaller and is modulated to a more intense signal of the respiratory activity. The measuring probe achieves high sensitivity to the pressure action, and these very weak heart signals are also recorded [59]. The FBG probe (described in more detail in Section 3.4) scans the mechanical action of the heart. Ballistocardiography falls into the category of non-invasive scanning of body movements. The movements of the body that are sensed by the sensor cause acceleration of blood that is spread inside the large vessels. From the medical point of view, the flow of blood hits the so-called aortic arch that is causing the movement of the body upwards and the subsequent movement of the body downwards when the blood descends [78]. From the historical point of view, this method has been known since 1877 (see [79]), but the first documented BCG signal measurement device was constructed by Isaac Starr in 1936. The current trend in the use of BCG signals for biomedical applications is rising again, thanks to new technologies, for example fibre-optic sensors (please see articles mentioned above). Figure 2 shows the waveform and comparison of ECG and BCG signals. H wave is a concave wave beginning near the origin of R wave, I wave is a small wave following H wave, J wave is the largest wave of the concave shape following immediately after I wave and K wave is a convex wave following J wave.

### 3.3. Non-Invasive Interferometric Measuring System

**Figure 3:**
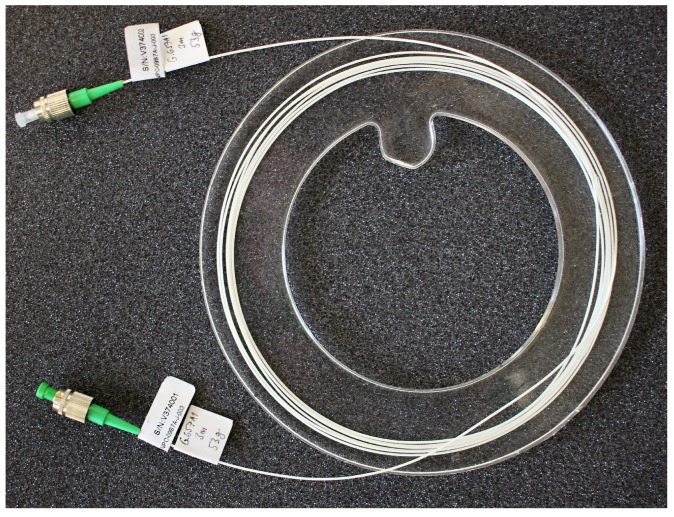
Fibre-optic interferometer measuring probe.

The interferometric measurement system uses the Mach–Zehnder layout, representing the basic two-wave interferometer. Optical radiation from a coherent 1550 nm source is fed into a 1 × 2 fibre splitter that divides it into measuring and reference optical paths. These parts are represented by two single-mode fibres having an equal length (3 m), wherein the reference part is located in a mechanically stable loop on the device, while the measuring loop is encapsulated in the PDMS coupling means to form a vibro-acoustically sensitive probe that can be placed on the human body (see Figure 3). The PDMS material was chosen after a series of experiments. The idea was to adopt the Young modulus to the body. The stiff materials such as plastics cannot be easily bent, thereby the human body must adapt shape to the sensor. On the contrary, too soft materials does not transfer vibrations very well. Good choices are rubber or silicone type materials, which can be easily bent and the contact with the skin is always good. The position was chosen to be in the middle of the chest even because the heart is usually located more on the left-hand side. Thus, we did not have to check where the person’s hearts are. The sensitivity of the designed sensors also allows sensors to be positioned i.e., on the abdomen (laboratory tested).

Bending does not affect sensing. In the case of the interferometer, the demodulation process (discussed below) contains parameters, which compensate the fibre attenuation. Variations in fibre attenuation can be suppressed using the bending insensitive fibres or by usage of lower wavelengths. In addition, the demodulation parameters can be recalculated over time and flexibly react to any unwanted attenuation.

The probe used is made using a 3 m optical fibre loop in a 900 μm tube having a radius of 5–8 cm and the total weight of the probe is 53 g. The first prototype probes were tested in a number of previous experiments [68], and, moreover, the sensitivity was so high that a similar system could be used for monitoring the foetus, as shown by the authors in [80].

The outputs from both optical paths merge into the second 3 × 3 coupling element forming the Mach–Zehnder interferometer, which translates the differences in phase delay into intensity changes, measurable by conventional power photodetectors. This coupling element has, in addition, three phase-shifted outputs whose output optical intensity can be described by Equation (5), where *C* is the mean value of the optical intensity, *A* is the amplitude of the intensity variation, and *m* is the index of the output port of the 3 × 3 fibre coupler assuming values of 1, 2 and 3. This allows easy passive demodulation of the phase difference between the interferometer arms. The passive homodyne demodulation method described e.g., in [81,82] can be used for demodulation:

(5) $$I_{m}\left(t\right)=C_{m}+A_{m}\cos\left[\Delta \phi \left(t\right)+\frac{2\pi }{3}\left(m-1\right)\right].$$

**Figure 4:**
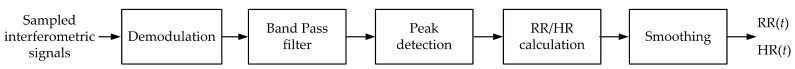
The signal processing from the FOI probe to determine the respiratory rate (RR) and the heart rate (HR).

The signal from the photodetectors is sampled by the National Instruments 9220 measuring module (Austin, TX, USA) in the cDAQ-9171 chassis, which is supported in the LabVIEW development environment, which was used to create a custom application that performed data recording, demodulation according to the above-stated technique, filtration, and RR and HR extraction functions. Additional signals were also connected to the same measurement module to enable synchronous data acquisition. The signal processing from the FOI probe to determine the respiratory rate (RR) and the heart rate (HR) is shown in Figure 4.

To determine the RR, a band pass filter of 0 to 0.5 Hz was used, wherein the upper cut-off frequency can be increased up to 5 Hz. To remove the long-term trend (especially the effect of temperature), a lower cut-off frequency, e.g., 0.1 Hz, can be introduced to remove this trend. A bandpass filter of 20 to 80 Hz was designed for HR determination, wherein the filter frequencies are based on the knowledge of the FOI sensors found in [83]. The upper cut-off frequency was further reduced to 50 Hz based on the findings from the initial MR measurements (see Section 4.1). The filter type used was the third order Butterworth. Peaks are detected above this signal. Based on their time marks, the respiratory rate (RR) is calculated according to the following formula: $RR=60/(t_{n}-t_{n-1})$, where $t_{n}$ is the time mark of the *n*-th peak and $t_{n}-1$ is the time mark of the preceding peak. The next step is to smooth the respiratory rate curve. A median filter with window size 3, which is statistically more resistant to remote observations than the moving average, is used for smoothing. The heart rate is calculated using the following relation $HR=60/(t_{n}-t_{n-1})$. A median filter with window size 7 is used for smoothing the heart rate over time.

### 3.4. Non-Invasive FBG Measuring System

**Figure 5:**
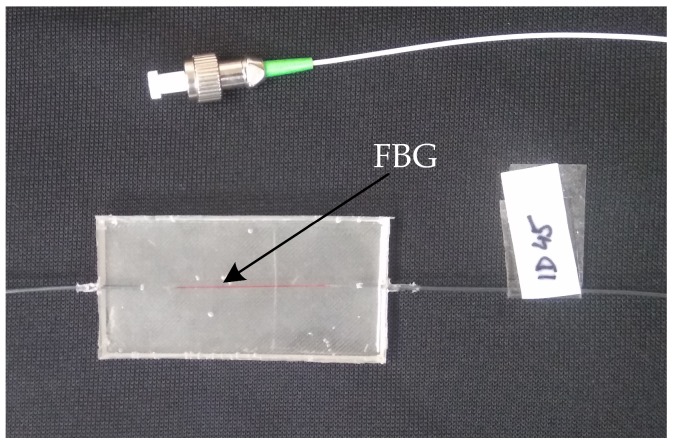
Fiber Bragg grating measuring probe.

The probe is encapsulated in the PMDS polymer. The results presented in [3] indicate that these type of encapsulation does not affect the structure and function of the FBG. In the case of the FBG probe being ideal to place a sensor close to the heart, because FBG in principle records body movements (chest/heart), please see Section 3.2. Bending of the feed fibre does not affect sensing because FBGs are spectral sensors where the absolute wavelength changes are evaluated. A photograph of the FBG probe prototype is shown in Figure 5. The size of the probe is 60×30×3 mm, the weight is 5 g, the shape is also adjusted with respect to the location in the rectangular clamping contact strip.

**Figure 6:**
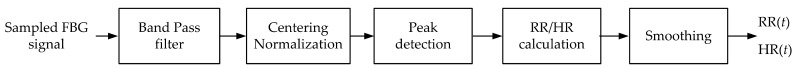
The signal processing from the FBG probe to determine the RR and the heart rate HR.

To determine the respiratory and heart rate, the signal from the FBG probe is processed according to Figure 6. Unwanted signal noise (motion artifacts, muscular activity) is produced by higher frequencies that are filtered out. To determine RR, a third order bandpass filter with cut-off frequencies from 0 to 0.5 Hz was used. As in the case of FOI, the trend may be eliminated by changing the lower cut-off frequency, e.g., the hyperventilation states can be recorded due to the temperature change of the probe and the change of the upper cut-off frequency. Furthermore, normalization and centring of the signal to the zero mean value are performed. Peaks are further detected above this signal, followed by the same signal processing to obtain the RR, which is described in detail in Section 3.3.

A bandpass filter with a cut-off frequency from 5 to 20 Hz was used to acquire the heart rate. It is selected for more efficient processing so that the output signal was made up of higher harmonics [59]. The signal filtered in such a way then provides a more reliable representation of the cardiac activity. Furthermore, the signal is centred and normalized. Peaks are detected above this signal, followed by the same signal processing to obtain the heart rate, which is described in detail in Section 3.3.

### 3.5. Reference Signals

The reference RR signal was obtained using PERT. The advantage of using this method is the same method of respiration monitoring as with optic fibre probes based on changes in the thoracic circumference associated with respiration. The Pneumotrace II belt made by UFI (Champaign, IL, USA) was used [84]. This belt was also used to fasten the FBG and FOI probes. Since typical output signals reach only 20–50 mV, an electrical amplifier was also inserted between the belt and the analog digital converter, which amplified the signal to the level of several Volts for better utilization of the ADC resolution. This was the above-mentioned module with converters made by National Instruments, specifically the NI-9220 module in the cDAQ-9171 chassis forming the measuring card.

The reference HR signal was obtained similarly. The reference ECG signal was acquired with the use of standard gel electrodes fixed to the tested person’s chest using a real-time monitoring system for an ECG signal with LIFEPAK 15 (Redmond, WA, USA) [85]. The ECG can be directly sampled using the analogue ECG output cable connected to the measuring module. This allowed simultaneous and synchronous sampling of data from all sensors at the same time for the purpose of the comparative study.

### 3.6. Schematic Diagram of the Measurement

**Figure 7:**
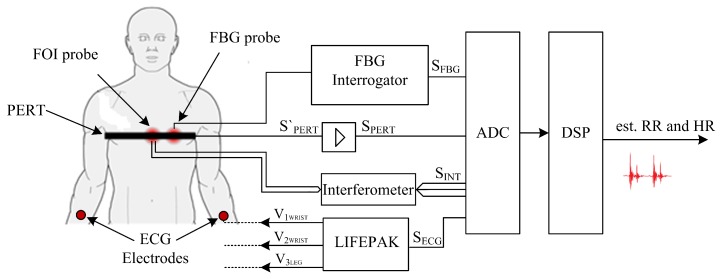
Basic schematic diagram of the measurement.

The basic schematic diagram of the measurement with non-invasive FOI and FBG probes, including the location of the reference ECG and PERT and processing of the signal, is shown in Figure 7. Electrodes were placed on the volunteers’ bodies and were connected to the LIFEPAK device.

The ECG output signal, referred to as $S_{ECG}$, is brought to the analog-to-digital converter (ADC). Furthermore, a fibre probe (FOI probe), which was part of the inteferometer whose outputs $S_{INT}$ were also connected to the ADC, was placed on the chest area. The second optic fibre probe (FBG probe) was placed next to this probe and connected to the FBG spectral interrogator unit with superluminescent diode (SLED) at center wavelength 1549.2 nm and an output power of 1 mW; the output from the $S_{FBG}$ interrogator was connected to the measuring module as well. Both probes were fastened to the body using an elastic band, which included PERT. The electrical output from the $S_{PERT}^{\prime }$ band was amplified into a $S_{PERT}$ form and was sampled by the same measuring card. The sampling frequency used was 100 kS/s/ch due to the requirement for minimal distortion of the amplitude modulation produced by the FOI system. A custom application programmed in LabVIEW 2017 (National Instruments, Austin, TX, USA) and selected scripts programmed in MATLAB (version 2017a) (MathWorks, Natick, MA, USA) were used for data recording and processing.

## 4. Results

The initial measurement with the goal to verify the basic functional assumptions of the measuring systems and probes in the MR environment was performed on four subjects (two males and two females) during regular examinations, upon their written contest. The subjects tested were between 24 and 47 years of age, their weight was between 45 and 92 kg and their height was between 157 and 186 cm. This measurement is described in more detail in Section 4.1 and was necessary because, although there are studies that describe practical measurements with fibre-optic sensors in MR scanner, these studies involve sensors of different constructions with a different implementation method on the human body, or by the method the information is evaluated and processed [41,45,46,86,87,88,89,90,91,92,93,94].

The follow-up long-term comparative laboratory measurement was conducted on a group of ten healthy male adults (hereinafter referred to as M1-M10) and ten female adults (hereinafter referred to as F1-F10), upon their written contest. The subjects tested were between 21 and 53 years of age, their weight was between 47 and 117 kg and their height was between 148 and 197 cm. No significant differences were found in the quality of the signal received depending on the age, weight, and height. The aim of the measurements performed was to obtain continuous records from the FOI probe, the FBG probe and the reference PERT and ECG devices. Long time series were evaluated, a total of 1194 min of experimental measurement records for respiratory rate measurements and 1197 min of experimental measurement records for heart rate measurements were analyzed.

The subjects were tested from the point of view of the laboratory experimental measurements performed in the supine and sitting positions. The experiments were discussed with a senior doctor and with a radiology assistant. Based on this consultation, all twenty test subjects were asked, during the experiments, to simulate their natural behaviour in the most accurate way (for instance, the focus was on the use of fine motor skills—minor movements of hands and arms, feet, legs, coughing, shallow and deep breathing, etc.). All of these aspects are taken into account in the results described above regarding the efficiency of both probes. The goal was to record measurements that would simulate, as closely as possible, the magnetic resonance environment and the environment for long-term recumbent patients. Based on the results, no significant differences were found in the quality of the signal received depending on the body position or minor artifacts such as a coughing, shaking hands, head movement, etc. A graphical illustration of the effect of the above-described body movements (minor effects), as well as the effect of body movement in full extent or walking (major effect) on the signal measured obtained by fibre-optic sensors, is shown in Section 4.1.

**Figure 8:**
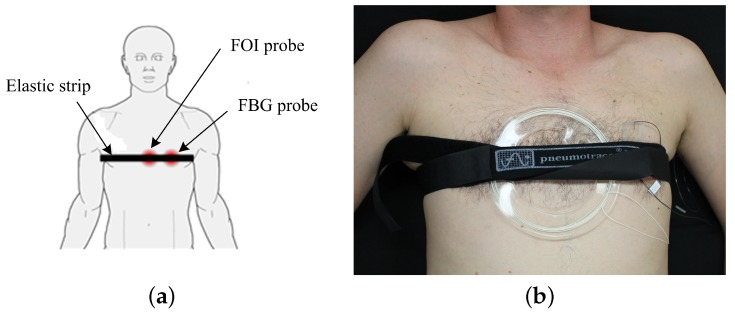
(**a**) a schematic location of the fibre-optic probes on the human body; (**b**) a photo of the location of the fibre-optic probes on subject M1 in the supine body position.

Both FOI and FBG probes were placed around the pulmonic area on the test subject’s chest and fixed into the position by a contact elastic band which was part of the PERT (please see Figure 8a,b, which shows a photograph of the comparative laboratory measurements performed, when subject M1 was in the supine position). During the implementation, the persons being measured did not declare any restriction of their comfort due to the elastic band used.

The B–A method [95], based on assessment of the differences between the utilized methods, was used for objective processing of the results. The differences between the methods, depending on the averages of these methods, are plotted in the graph. This differential chart allows assessment of whether the differences are of a systematic nature (whether the difference is systematically different from zero) and how much the differences vary (their dispersion or standard deviation). The reproducibility is considered to be good if 95% of the results lie within a ±1.96 SD (Standard Deviation) range.

### 4.1. The First Phase of the Comparative Study

In the first phase of the comparative study, a pilot measurement was carried out in the MR environment to verify the functionality of the measuring systems and the probes designed. The aim was to gain knowledge on the appropriate placement of sensors on the test subjects, installation of fibre-optic lead-in cables, and information on whether the probes designed can be used to measure the vital functions of the human body during an MR examination. During the examination, there are strong acoustic shocks and subtle mechanical movements of the device and it is necessary to verify whether the signal obtained from the fibre-optic probes will not be degraded by these artifacts. The measurements were performed at a private clinic in Prostějov, under the supervision of a senior radiology assistant. The type of the MR scanner used in the experiment was Signa HDxt 1.5T (GE Healthcare, Chicago, IL, USA) [96]. The MR scanner is equipped by the manufacturer with the ability to monitor the heart rate using a PPG sensor and a RR measuring device in the form of a pneumatic sensor. Unfortunately, the values obtained by these sensors cannot be used in our case as a suitable reference since they are only informative values, in the form of visual waveforms on the radiology assistant’s monitor, that cannot be extracted from the device and synchronized with the outputs of the fibre-optic system being developed.

**Figure 9:**
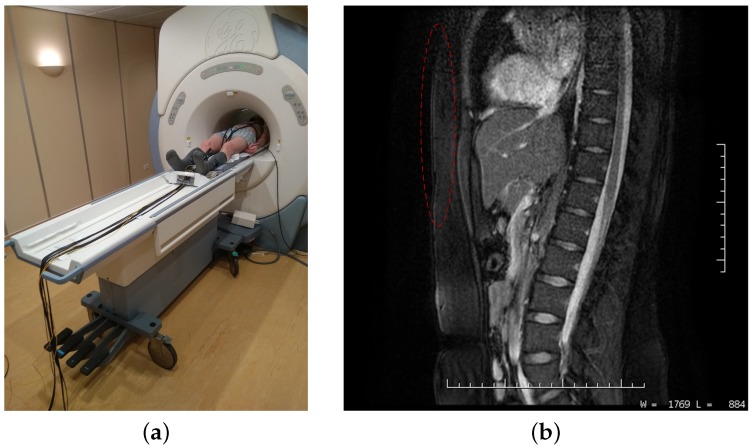
(**a**) Photography from the MRI examination; (**b**) MRI image acquired from the sensor location: sagittal view—test subject M2 (512 × 512 resolution).

The waveforms obtained from the fibre-optic sensors were subjectively compared to the above-stated values from the MR scanner and consulted with a radiology assistant, who confirmed our initial assumptions about the functionality of the measuring systems and probes designed. No negative effects on the MR scanner operation were observed during the examinations performed (both types of probes are not visible in the pictures, i.e., the record is not compromised (see Figure 9)—the location of the fibre-optic probes is marked in red colour). Continuous recordings of the vital functions from both types of probes during all procedures performed were made during the examination.

**Figure 10:**
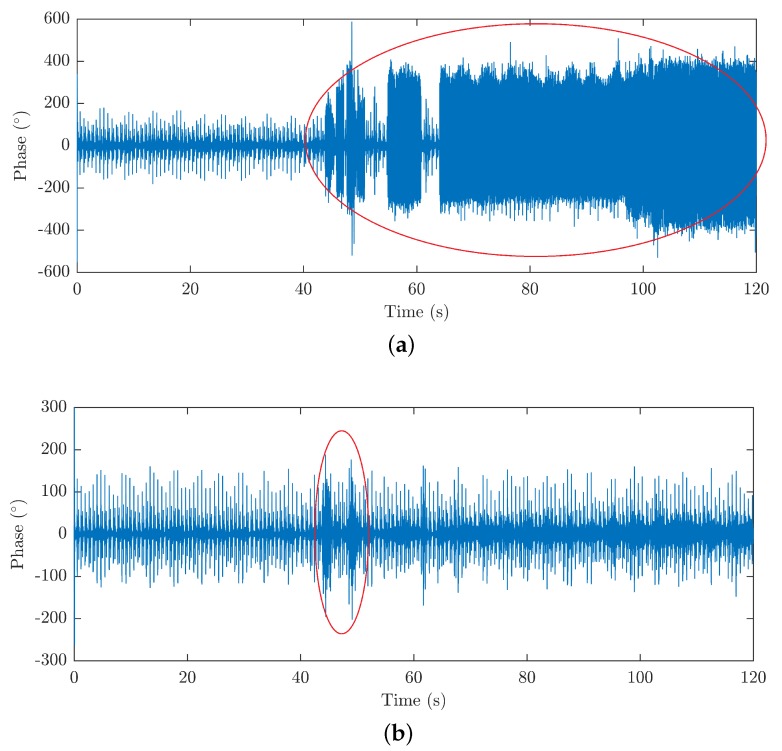
(**a**) Example of a FOI signal from the MRI during the examination; (**b**) suppression of undesirable effects by filtration.

The experimental measurement performed also confirmed that acoustic signals that distorted the HR measurement in the FOI system proved to be the dominant phenomenon. No artifacts were seen during the measurements on the FBG system using the FBG probe or for determination of the RR on both types of the sensory systems (the FBG probe and the FOI probe). Figure 10a shows a signal from the FOI filtered only by the upper pass filter with a cut-off frequency of 20 Hz corresponding to the interferometric PCG from which the HR can be determined.

Measurements of typical MR acoustic frequencies can be found in the literature, wherein, for example, the authors of the study of comparisons of individual types of resonances [97] report dominant acoustic peaks in the signal around the frequencies of 70, 300, 550 and 1100 Hz for GE Signa 1.5T magnetic resonance (GE Healthcare, Chicago, IL, USA), which, due to its magnetic performance, corresponds to the newer HDxt model used during our measurements. The other types of magnetic resonances mentioned in the publication showed interference in a similar frequency range, typically 50 to 1000 Hz. These findings were confirmed as artefacts caused by the MR operation (Figure 10a) completely disappeared from the signal by limiting the frequency filter for interferometric PCG from 20–80 Hz to 20–50 Hz and only minor motion artifacts remained (see Figure 10b).

In future, another possible way could comprise implementation of probes into the MR system, where a second FOI probe that would provide a reference differential signal for compensation of the acoustic noise generated during device operation could be placed inside the MR scanner. The measurement findings also demonstrate the ability of the FOI probe to read acoustic signals, so they could also be used as a microphone.

The follow-up measurements were moved to the laboratory environment due to the large number of test subjects and the inability to use the reference signals directly from the sensors in the MR scanner. The goal of the laboratory study was to perform measurements that would simulate as closely as possible the harsh MR environment and the environment of long-term health care facilities, hospitals and clinics.

### 4.2. Comparative Measurement of Vital Functions in Laboratory Conditions

#### 4.2.1. Respiratory Rate Measurement

**Figure 11:**
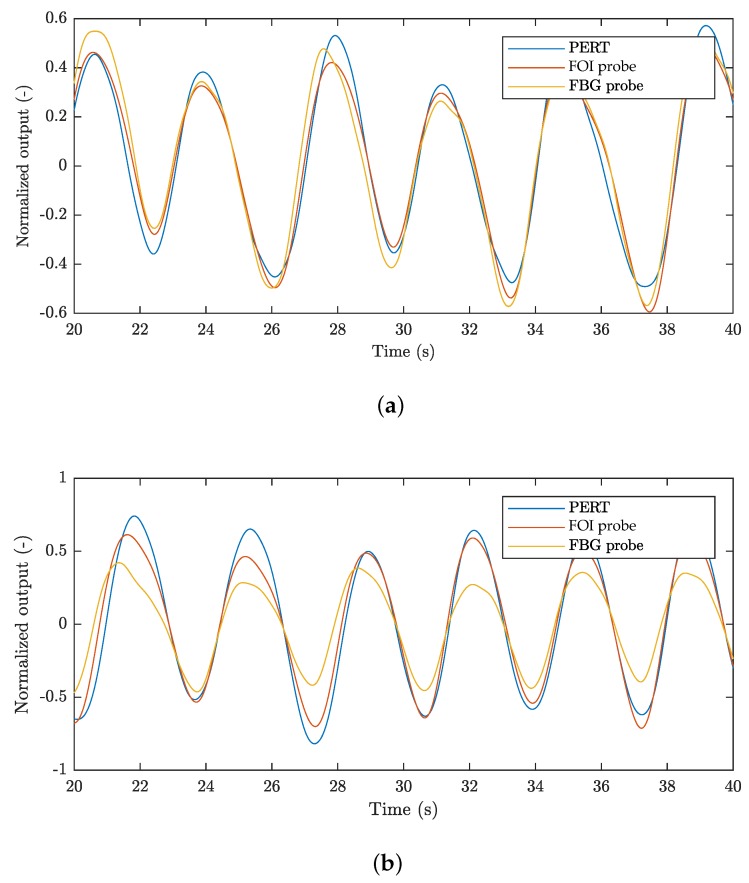
(**a**) an example of filtered signals representing the respiratory activity of subject M1; (**b**) an example of filtered signals representing the respiratory activity of subject F1.

This section provides a study focused on respiratory rate monitoring of the above-stated 20 test subjects. Figure 11a shows a short example (20 s) of filtered signals, representing the respiratory activity from both of the fibre-optic sensors and the reference signal, measured on subject M1. Figure 11b illustrates a similar example of respiratory activity signals of subject F1.

The signals were normalized due to different units on the vertical axis. The congruence of the period of both of the signals from the fibre-optic sensors with the reference signal is clearly visible. The reference signal is principally inappropriate for determining the lung volume and the same applies to the signals from the fibre-optic probes being compared which cannot be compared in terms of the absolute amplitude. Despite this, there is good congruence of all three signals, especially those from the probes that we devised. If lung volume measurements are needed, it would be necessary to use a different reference method as well as fibre-optic sensors of a different construction.

**Figure 12:**
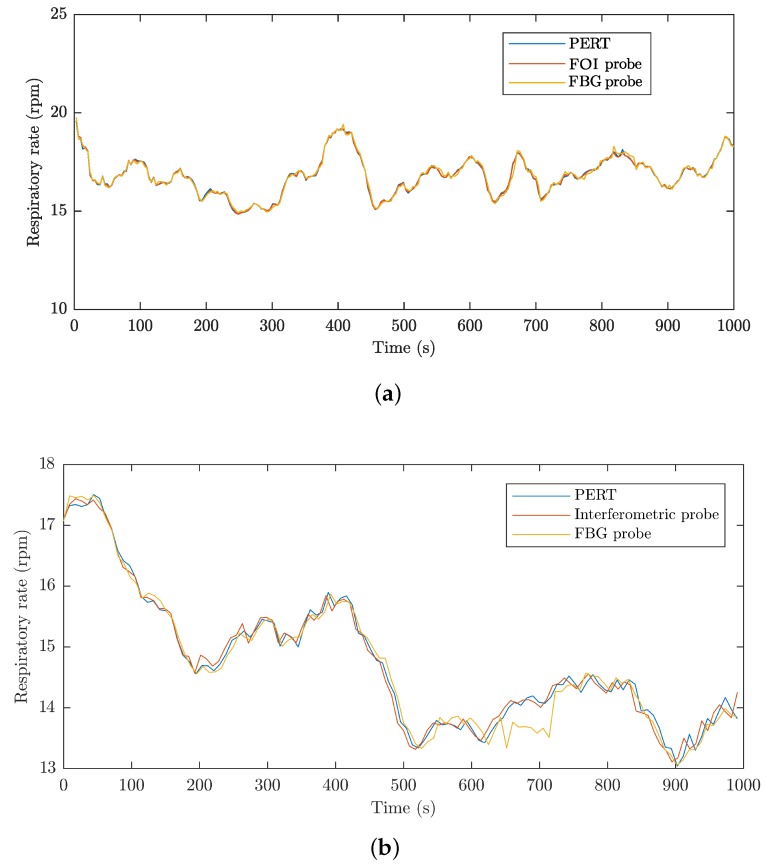
(**a**) an example of the RR waveform for subject M1; (**b**) an example of the RR waveform for subject F1.

The RR can be subsequently calculated from the filtered respiratory activity signals Figure 12a shows an example of the RR waveform for subject M1, and Figure 12b is a similar example of RR waveform for subject F1. RR waveforms are expressed in rpm (respirations per minute). The waveforms represent 1000 s of the signal recorded. It can be seen from the graphs that the signals from the probes designed by us correlate with the signal from PERT, wherein, in the case of the M group subjects, the signals from the probes generally correspond more than in the case of the F group subjects. This difference is probably caused by the different physiological constitution of the subjects of the groups.

**Figure 13:**
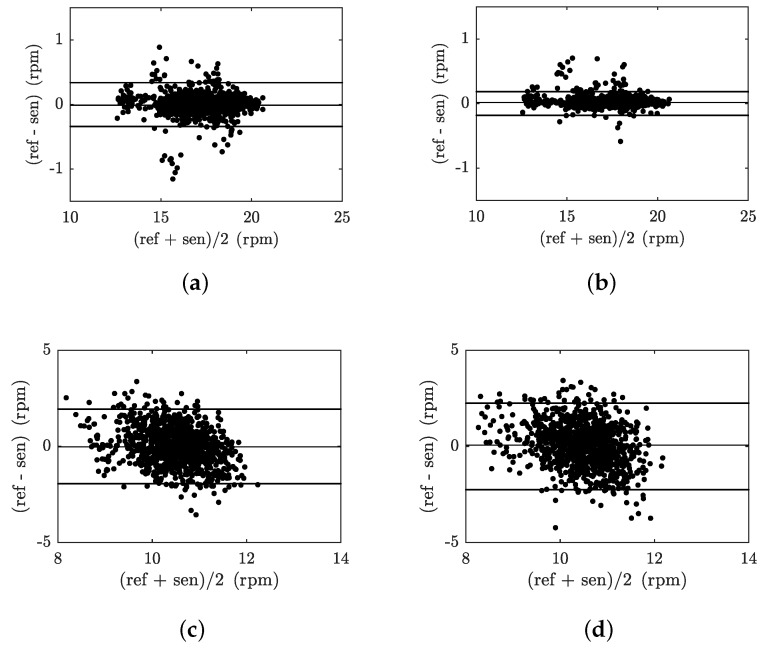
(**a**) B–A plot of M1 subject—FBG probe vs. PERT; (**b**) B–A plot of M1 subject—FOI probe vs. PERT; (**c**) B–A plot of F1 subject—FBG probe vs. PERT; (**d**) B–A plot of F1 subject—FOI probe vs. PERT.

Figure 13 illustrates examples of B–A graphs for the above-stated 1000 s recordings representing the respiratory activity of subjects M1 and F1. Figure 13a represents the B–A plot for the FBG probe and the reference PERT for subject M1, Figure 13b represents the B–A plot for the FOI probe and the reference PERT for subject M1, Figure 13c represents the B–A plot for the FBG probe and the reference PERT for subject F1 and Figure 13d represents the B–A plot for the FOI probe and the reference PERT for subject F1. The differences between the probe and the reference traces $\left(x_{1}-x_{2}\right)$ are plotted against the average $(x_{1}+x_{2})/2$. A number of characteristic features can be derived from the B–A graphs at first glance. From the differences shown, it is obvious that the they are approximately symmetrically divided and there are no statistically significant differences between the data analyzed from the fibre-optic probes and the PERT reference (see Table 3).

Table 3 describes the overall evaluation of the experimental RR measurements for all 20 test subjects, including the statistical evaluation within the B–A analysis. Time(s) represents the total measurement time for the subject, the ARR is expressed in respirations per minute (rpm) and represents the average respiratory rate of the subject throughout the measurement. NoS represents the number of samples measured by FBG and FOI probes, Error means the absolute number of samples that are outside the ±1.96 Standard deviation (SD) range and Rel. Error indicates the number of defective samples relative to the total number of samples expressed in percent. For the entire data set (all 20 test subjects), 95.53% of the values lie within the ±1.96 SD range in RR determination for the FBG probe. In the case of FOI probe, for the entire data set (all 20 test subjects), 95.66% of the values lie within the ±1.96 SD range in RR determination. It is obvious that the FOI probe offers slightly higher accuracy (0.13%) in terms of respiratory rate measurement (based on the B–A analysis). The relative error reaches 4.47% in the case of the FBG probe and 4.34% in the case of the FOI probe. Based on the results, it can be stated that no significant differences between individuals were observed. The represented results show no systematic errors, and the error has no proportional character and it does not depend on the RR value. The B–A statistical analysis demonstrates the RR detection with a satisfactory accuracy for multiple subjects for both the FBG and the FOI probe.

#### 4.2.2. Heart Rate Measurement

**Figure 14:**
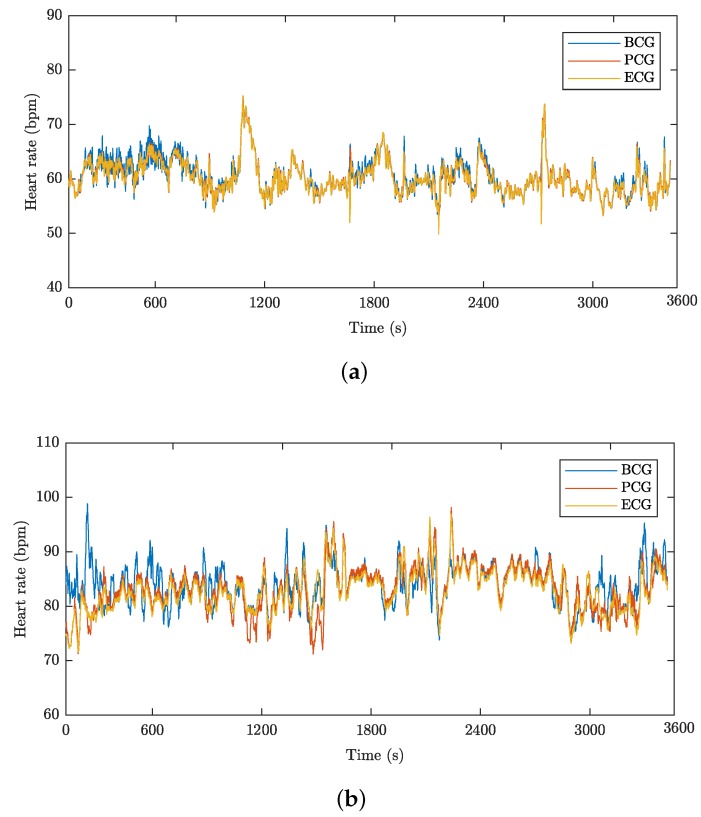
(**a**) The HR waveform for subject M1; (**b**) the HR waveform for subject F1.

This section provides a study focused on heart rate monitoring of the above-stated 20 test subjects. Figure 14a shows an example of a one-hour correlated HR recording of subject M1 representing the time waveform of cardiac activity obtained by measurement performed using a prototype of the FOI probe (labelled PCG) and the FBG probe (labelled BCG) together with an ECG reference (labelled ECG). Figure 14b shows a similar one-hour HR recording of subject F1. The heart rate waveforms are expressed in bpm (beats per minute).

**Figure 15:**
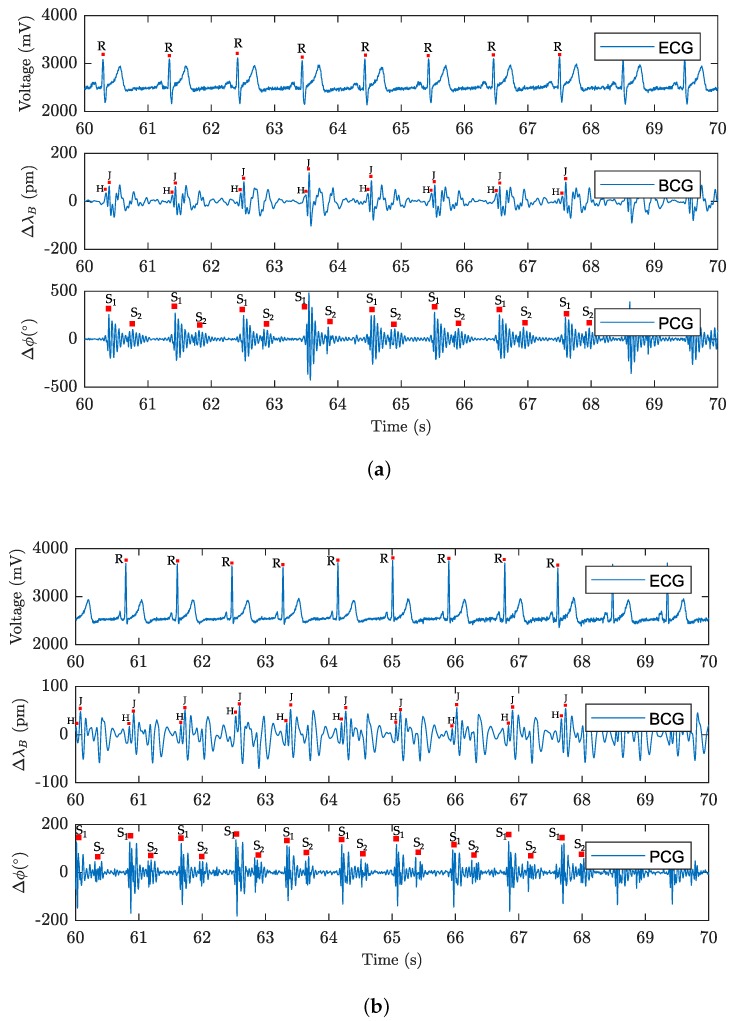
(**a**) An example of 10-s signals representing the cardiac activity of subject M1; (**b**) an example of 10-s signals representing the cardiac activity of subject F1.

Figure 15a,b with a 10-s recording are provided for a more detailed view of the signals representing the cardiac activity from both the fibre-optic sensors and the reference signal. The individual maxima detected in the ECG signal are characteristic of R wave, in the case of the BCG signal from the FBG probe, the individual maxima characterizing H wave and J wave are marked, and for the FOI producing the PCG signal, the main sounds $S_{1}$ and the auxiliary sounds caused by the closing of the semilunar valves $S_{2}$ are indicated, which is in line with the theory described in Section 3.

**Figure 16:**
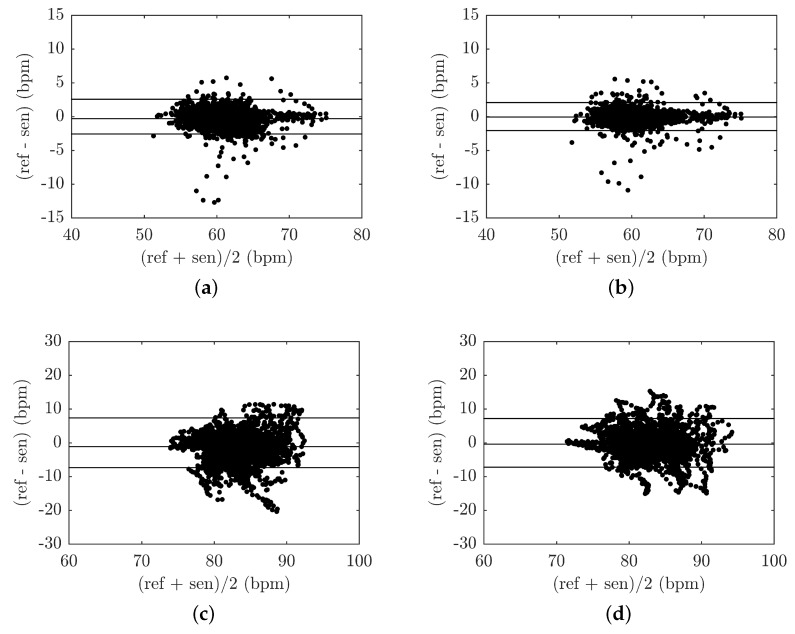
(**a**) B–A plot of M1 subject—FBG probe vs. ECG; (**b**) B–A plot of M1 subject—FOI probe vs. ECG; (**c**) B–A plot of F1 subject—FBG probe vs. ECG; (**d**) B–A plot of F1 subject—FOI probe vs. ECG.

Figure 16 shows B–A graphs for the above-stated one-hour recordings representing the cardiac activity of subjects M1 and F1. Figure 16a represents the B–A plot for the FBG probe and the reference ECG for subject M1, Figure 16b represents the B–A plot for the FOI probe and the reference ECG for subject M1, Figure 16c represents the B–A plot for the FBG probe and the reference ECG for subject F1 and Figure 16d represents the B–A plot for the PCG probe and the reference ECG for subject F1. The differences between the probe and the reference traces $\left(x_{1}-x_{2}\right)$ are plotted against the average, $(x_{1}+x_{2})/2$. From the B–A graphs shown, it can be interpreted that there are no statistically significant differences between the data analyzed from the fibre-optic probes and the references (see Table 4).

Table 4 describes the overall evaluation of the experimental heart rate measurements for all 20 test subjects, including the statistical evaluation within the B–A analysis. Time(s) represents the total measurement time for the subject, the AHR represents the average heart rate of the subjects, NoS represents the number of samples obtained by FBG and FOI sensors, Error means the absolute number of samples that are outside the ±1.96 Standard deviation (SD) range and Rel. Error indicates the number of faulty samples relative to the total number of samples expressed in percent. For the entire data set (all 20 test subjects), 95.23% of the values lie within the ±1.96 SD range in HR determination for the FBG probe. In the case of FOI probe, for the entire data set (all 20 test subjects), 96.22% of the values lie within the ±1.96 SD range in HR determination. It is obvious that the FOI probe offers higher accuracy (0.99%) in terms of heart rate measurement (based on the B–A). Based on the results, it can be stated that no significant differences between individuals were observed. The relative error is 4.77% in the case of the FBG probe and 3.78% in the case of the FOI probe. The represented results show no systematic errors, and the error has no proportional character and it also does not it depend on the HR value. The B–A statistical analysis demonstrates the HR detection with a satisfactory accuracy for multiple subjects for both the FBG and the FOI probe.

#### 4.2.3. The Influence of Motion and Acoustic Artifacts on the HR and RR Signals Measured

**Figure 17:**
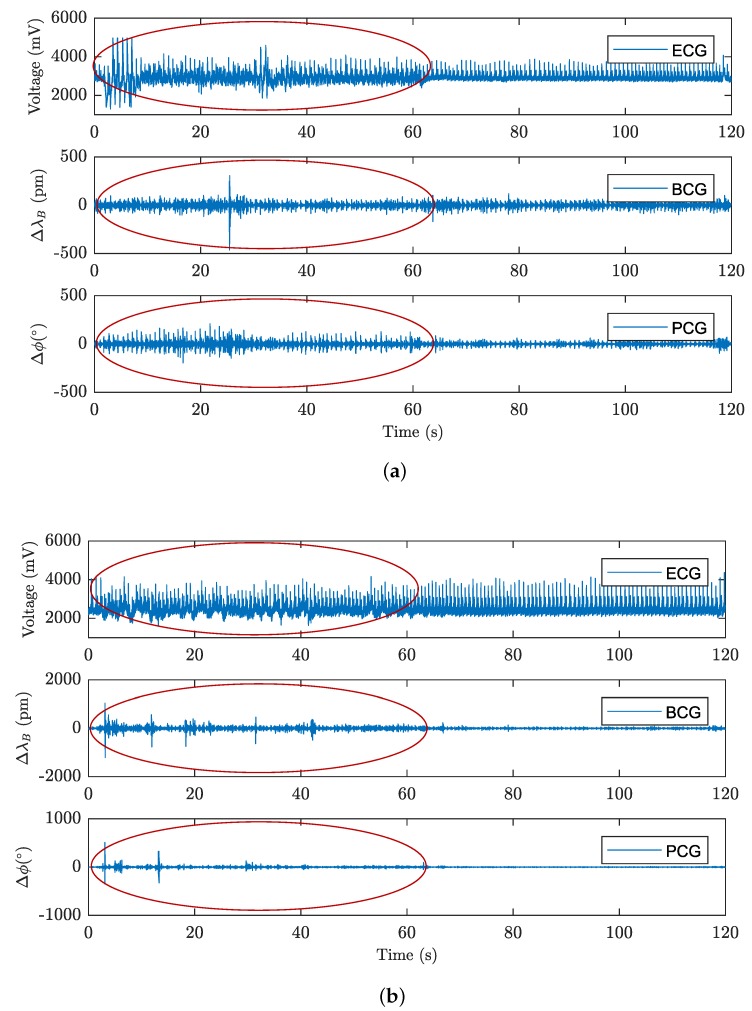
(**a**) an example of the impact of minor effects on the signal measured; (**b**) an example of the impact of major effects on the signal measured.

Before performing the above-stated comparative measurements, the first volunteers were asked to simulate motion or acoustic artifacts during the laboratory measurements performed. The goal was to verify their impact on measurements and to allow the test subjects to avoid unwanted motion artifacts. The motion artifacts analyzed included, for example, slight movement or trembling of the legs, slight hand movement, torso rotation, head movement, rapid breathing or coughing or snoring. Many of them, such as shallow breathing, hand movement, and head movement, did not distort the HR measurements to degrade the signal (see Figure 17a where a hand movement was selected to show a minor artifact), wherein, for this image, the person was asked to perform these random movements for 60 s and, then, to stay still. It is fairly obvious that these artifacts also caused slight distortion of the ECG signal because one of the ECG electrodes was placed on the hand.

Figure 17b shows the effect of major artifacts such as a continuous body movements to the fullest extent or walking. These movements seriously distorted the resulting BCG and PCG recordings and prevented correct HR determination. The advantage, however, is that these motion artifacts are detectable, which can be used, for example, for detecting sudden attacks occurring during the measurement or long-term monitoring of sleep quality. During the measurements, the test subjects were asked to remain in a rest position and to avoid major motion artifacts.

On the contrary, no motion artifact had an effect on the RR measurements, but, to determine the correct rate, it was necessary to shift the upper cut-off frequency of the band pass filter (up to 5 Hz) when processing the RR signal during rapid breathing. All of these minor movements and sound aspects are taken into account in the results described above regarding the efficiency of both probes. In the follow-up research, the authors are ready to carry out a detailed analysis of the influence of the major artifacts.

## 5. Discussion

The results have shown that both types of sensors can reliably monitor both the heart rate and the respiratory rate simultaneously using a single probe. Because the interferometric principle, which uses phase difference measurement between two waves, in contrast to measuring the wavelength of the reflected optical radiation, is generally a more sensitive principle, and this corresponds to the results achieved, where the interferometric probe (FOI) was able to provide higher accuracy (by 0.99% in the case of heart rate measurements, by 0.13% in the case of respiratory measurements, according to the B–A analysis).

The advantage of grating (FBG) sensors is the sensitivity of the probe only at the point of the optical grating, wherein the lead fibre is not sensitive to vibrations and other undesirable effects. The design of the sensor (probe) is extremely compact because the grating itself is inscribed in an optical fibre having a length <1 cm. Only one fibre can lead to the probe and it can contain a whole range of FBG sensors that can be used for monitoring at other sites or also other physiological quantities. Such a quasi-distributed system can measure a number of signals without interacting with each other through their shift in the spectral region.

The disadvantage is difficult evaluation of the output signal that has to be conducted spectrally, which, in principle, leads to a higher price for the resulting measuring system. Such a system uses expensive wide-spectrum sources, the power of which is spread over the entire spectrum, resulting in individual spectral components reflected by FBG sensors achieving low power and low signal-to-noise ratios (SNRs). This leads to higher demands on the detection capabilities of the evaluation unit.

Interferometric systems provide higher sensitivity, but this is not so necessary in this particular application, but the higher achievable accuracy of measurement is the advantage, as the results show. Another advantage is that, at the output, the interferometer produces amplitude modulation that can be detected by conventional photodetectors, a radiation source is a coherent laser diode, mass-produced and used in telecommunications, and the entire measuring system can thus be constructed from commonly available components, which are thus more affordable. Interferometric systems also have the potential for detecting acoustic signals (sounds) of frequencies higher than 80 Hz and they could be used, for example, for monitoring respiratory and arousal events.

In certain cases, the fact that the FOI probe requires a much longer section of the optical fibre (meters) and is not so compact can be considered to be disadvantageous. In addition, a reference loop of the same length must be placed in the device and, moreover, it must be isolated from ambient influences as both loops are sensitive to vibrations and other influencing phenomena. If there is a requirement for multi-point measurement conducted by interferometers, a problem with multiplexing the sensors arises. The first problem is the need for a higher number of lead-in fibres for each sensor, wherein this number increases linearly with the number of sensors when no wavelength division multiplex is used, which results in a more expensive and more complicated sensory system topology. This is related to another complication, since each sensor requires its own detection components.

The benefits of both types of the sensory systems include the possibility of remote evaluation of the quantities measured. The distance is limited by the type of the interconnecting optical fibre used located between the probes and the evaluation units, which is given by the fibre attenuation coefficient and, further, by the power and wavelength of the radiation source used. In addition, both types of systems provide a reduced patient load in the form of a smaller number of sensors placed, since multiple quantities can be monitored at the same time.

Sweat and temperature present no challenges to interferometric sensor operation. Sensors can be operated even with indirect contact with skin (i.e., over a T-shirt). Sweat as an extra layer does not introduce any issues. During the measurements, subjects were occasionally sweating so these phenomena are already included in the presented results. Temperature changes are apparent in the measured results with both sensors, nevertheless, the phenomena are effectively filtered out (please see Section 3.3 and not affecting presented results. In case of grating sensors, of course, these are dependent on ambient temperature and temperature of patient. However, signals of HR and RR have fast dynamic character; therefore, slow changes of temperature or sweating have no influence on evaluation accuracy of HR and RR of human body.

Basically, any fibre interferometric systems can be used. Out of the four most known arrangements (Mach–Zehnder (MZI), Michelson, Sagnac, Fabry–Perot (FPI)), the MZI was chosen as the sensor can be cheapest. A slight improvement can bring the Michelson interferometer since it will require single lead fibre to the sensor. FPI can bring the desired sensor compactness; however, the sensitivity would be very different from the two before mentioned interferometer configurations. FPI is also very sensitive to the source wavelength changes; therefore, precise temperature and current controller must be used in combination with thermoelectric cooler (TEC) compatible laser packaging. Authors [70,71,72] presented a Fabry–Perot (FP) interferometer inside the plastic tube; Respiratory Rate tests were carried out using a simulated breathing flow system, with relation to thin-film refractive indices and thickness; but no data on sensor efficiency were presented.

Measurement of HR and RR performed by FBG probe can be theoretically influenced by the light source due to imperfect thermal stabilization of broadband optical light sources causing changes of optical output power and shape of the spectral distribution of light, which can influence the shape of reflected spectra from FBG and therefore influence evaluation of Bragg wavelength shift. These mentioned changes, fortunately, have slow behaviour in comparison to HR and RR and therefore can be neglected.

Used optical sources (for FBG as well as for FOI system) were chosen at 1550 nm because the used silica optical fibres (in case of both probe types) have lowest attenuation and photodetectors’ highest sensitivity at this wavelength. A wavelength of 1550 nm was also chosen with respect to lower construction costs as telecommunication optical fibres offer low-cost waveguides in comparison with fibres for different wavelengths. In addition, in the case of the FBG system, this wavelength allows direct connection into the hospital optical network infrastructure and therefore the possibility of remote processing and evaluation of data measured by probes.

Compared to the results achieved by other studies, the sensory system devised by us obtains similar results. The study in the article [46] that uses the FBG sensor achieves slightly higher accuracy when determining the HR (95.89%) and the RR (96.10%) relative to the reference signal. This may be due to both the different design of the sensor and the fact that the evaluation was performed on only 12 subjects with a recording length of only 5 min per person. Motion artifacts may not have manifested themselves sufficiently in such a short time. A similar study on 10 subjects was also provided by the authors of the article [28] who devised an interferometric sensor that was incorporated into a mattress but was not designed for the MR environment. Nevertheless, thanks to the nature of the fibre-optic sensors, it is possible to assume the possibility of its use even in the MR environment. The results obtained showed a slightly lower sensitivity in determining the RR (95.3%), while, when determining the HR, the situation was the opposite (99.4%). The study was performed on 2 min recordings. Dziuda et al. [41] presented an overview article that aims to systematize the knowledge of fibre-optic techniques for recording life functions and to indicate the current directions of development in this area. A total of 47 MR-tested or potentially MR-compatible sensors have been described, but no sensor has been encapsulated in the PDMS polymer. A similar overview article was presented by Quandt et al. [98] focusing on body monitoring and health supervision by means of optical fibre-based sensing systems in medical textiles.

The measuring probes presented in this article are relevant or partly relevant to the following patent documents: a fibre-optic measuring system for monitoring the vital functions of the human body (document number: 306857) and a device for monitoring vital functions of a pregnant woman’s foetus (document number: 307183).

## 6. Conclusions

Fibre-optic sensory systems are increasingly applied in the biomedical industry and are a suitable and affordable alternative to commonly used devices in terms of HR and RR determination. The authors of the publication perceive the applicability of the systems and probes designed referred to in the article as provisional, primarily in the MR environment or for patients with a minimum motion load (long-term ill patients). This was the reason why this publication, which provides a comparative study of two fibre-optic non-invasive sensory systems relative to a conventional ECG or a Piezo-Electric Respiration Transducer in the application of human body HR or RR monitoring, has been compiled.

An extensive study, based on 20 test subjects with a total recording length of 1194 min for the RR measurement and 1197 min for the HR measurement, was conducted. None of the test subjects who participated in the study complained about any discomfort associated with the presence of the sensors placed on their thorax. The data acquired was compared by the objective B–A method. In the case of the RR determination for both types of the presented sensors, more than 95% of the values lie within the ±1.96 SD range (95.66% for the FOI probe and 95.53% for the FBG probe). In the case of the HR determination for both types of the presented sensors, again, more than 95% of the values lie within the ±1.96 SD range (96.22% for the FOI probe and 95.23% for the FBG probe). Based on a comparison of the study results, it can be concluded that the FOI probe offers higher accuracy for the measurements of human body RR as well as HR. The relative error level during the RR and HR measurements in the case of both of the fibre-optic sensory systems has not exceeded 5% and is, therefore, acceptable to clinicians because the systems designed are primarily intended for monitoring rather than diagnosis.

During their verification, the outcomes of this work captivated the attention of clinicians at the University Hospital (Ostrava, Czech Republic). For this reason, as a next step, the authors of the publication plan to establish closer cooperation with the doctors, since the clinicians intend to use these novel sensors in the near future to perform a long-term study on hyperventilation aspects during MR examinations.

## Figures and Tables

**Table 1:** A comparison of both fibre-optic systems.

	Advantages	Disadvantages	Application Possibilities	Cost (Approximately) Probe/Evaluation Unit
FBG system	the feed fibre is not sensitive to vibrations and other undesirable effects; one lead-in fibre for sensor; design of the sensor (probe) is extremely compact = small size; the possibility of quasi-distributed measurement - sensors can be multiplexed on a single optical fibre with single evaluation unit; the possibility of remote evaluation (hundreds meters); MRI-compatible	costly spectral evaluation (higher cost); low signal-to-noise ratio (in the case of multiple probes)	RR and HR of the human body	100 $/18,000 $
FOI system	higher sensitivity; measuring system can thus be constructed from commonly available components (low-cost); the potential for detecting acoustic signals (monitoring respiratory and arousal events); MRI-compatible	sensor’s dimensions (potentially discomfort); storing of the reference arm; a higher number of lead-in fibres for each sensor	RR and HR of the human body; monitoring respiratory and arousal events	200 $/4000 $

**Table 2:** A comparison of the most interesting fibre-optic sensors (based on the both interferometric and Bragg gratings designs) designed for monitoring respiratory rate and heart rate.

**Interferometric Sensors**		
**Reference**	**Type of Interferometer, Measured Parameter**	**Quantitative Data on Sensor Efficiency**
[65,66] (Patents)	Mach–Zehnder, Michelson, Sagnac, Fabry–Perot (RR and HR)	No efficiency data presented by authors
[37,67,68]	Mach–Zehnder, Michelson, Sagnac, Fabry–Perot (RR and HR)	Insufficient data, our previously research: No efficiency data presented by authors
[33,34,35,36,69]	Michelson (RR and HR)	Most recent, experimental study: recording time: 60 s; sensitivity: 97.64 ± 7.28%, precision: 99.38 ± 2.80%, maximum relative error: 7.35 ± 7.20% for RR detection, sensitivity: 99.46 ± 1.11%, precision: 99.60 ± 1.05%, maximum relative error: 3.16 ± 2.32% for HR detection
[27]	Speckle interferometer (RR)	Sensitivity: 98.4 ± 1.1%, precision: 98.2 ± 2%
[38]	Micro tapered Mach–Zehnder (RR)	Insufficient data
[70,71,72]	Fabry–Perot interferometer (RR)	No efficiency data presented by authors
**Fibre Bragg Grating Sensors**		
**Reference**	**Measured Parameter**	**Quantitative Data on Sensor Efficiency**
[39,40,41,42,43,44,45,46,47,48,49,50,51,52,53,54,55,56,57,58,59,60,61]	RR and/or HR	Most recent experimental study: An RMS (root mean square) value of the relative error is below 1.8%, our previously research (2 FBGs): maximum relative error 5.41% for RR detection, sensitivity 96.54% for HR detection

**Table 3:** Summary of respiratory measurements.

			FBG Probe				Interferometric Probe			
Sub.	Time (s)	ARR (rpm)	NoS (-)	Error (-)	Rel. Error (%)	Samples in ±1.96 SD (%)	NoS (-)	Error (-)	Rel. Error (%)	Samples in ±1.96 SD (%)
M1	3594	17	1025	45	4.39	95.61	1026	45	4.39	95.61
M2	3557	16	957	41	4.28	95.72	957	40	4.18	95.82
M3	3567	18	1057	39	3.69	96.31	1057	38	3.60	96.40
M4	3612	17	1031	38	3.69	96.31	1031	37	3.59	96.41
M5	3512	15	869	41	4.72	95.28	870	40	4.60	95.40
M6	3517	16	939	37	3.94	96.06	939	37	3.94	96.06
M7	3587	15	898	35	3.90	96.10	899	35	3.89	96.11
M8	3574	17	1014	41	4.04	95.96	1015	40	3.94	96.06
M9	3547	15	889	36	4.05	95.95	889	36	4.05	95.95
M10	3568	18	1069	42	3.93	96.07	1071	42	3.92	96.0
F1	3727	15	922	59	6.40	93.60	923	51	5.53	94.47
F2	3624	11	667	37	5.55	94.45	667	35	5.25	94.75
F3	3498	13	761	38	4.99	95.01	762	36	4.72	95.28
F4	3614	12	719	34	4.73	95.27	719	34	4.73	95.27
F5	3608	15	904	39	4.31	95.69	904	39	4.31	95.69
F6	3578	14	835	33	3.95	96.05	836	31	3.71	96.29
F7	3595	14	841	37	4.40	95.60	842	37	4.39	95.61
F8	3587	15	898	42	4.68	95.32	898	41	4.57	95.43
F9	3574	16	954	45	4.72	95.28	955	43	4.50	95.50
F10	3604	14	842	43	5.11	94.89	842	42	4.99	95.01
Sum	71,644		18,091	802	4.47	95.53	18102	779	4.34	95.66

**Table 4:** Summary of heart rate measurements.

			FBG Probe				FOI Probe			
Subject	Time (s)	AHR (bpm)	NoS (-)	Error (-)	Rel. Error (%)	Samples in ±1.96 SD (%)	NoS (-)	Error (-)	Rel. Error (%)	Samples in ±1.96 SD (%)
M1	3602	63	3781	171	4.52	95.48	3781	121	3.20	96.80
M2	3547	76	4487	197	4.39	95.61	4489	165	3.68	96.32
M3	3628	77	4664	208	4.46	95.54	4665	158	3.39	96.61
M4	3571	62	3691	185	5.01	94.99	3692	133	3.60	96.40
M5	3498	76	4422	188	4.25	95.75	4422	159	3.60	96.40
M6	3521	75	4398	185	4.21	95.79	4399	157	3.57	96.43
M7	3617	71	4286	169	3.94	96.06	4288	149	3.47	96.53
M8	3647	68	4128	168	4.07	95.93	4131	152	3.68	96.32
M9	3645	66	4028	171	4.25	95.75	4029	147	3.65	96.35
M10	3589	73	4382	185	4.22	95.78	4384	158	3.60	96.40
F1	3507	82	4789	281	5.87	94.13	4781	187	3.91	96.09
F2	3661	81	4941	266	5.38	94.62	4943	189	3.82	96.18
F3	3586	87	5190	275	5.30	94.70	5192	204	3.93	96.07
F4	3608	85	5118	249	4.87	95.13	5128	189	3.69	96.31
F5	3591	82	4905	258	5.26	94.74	4905	197	4.02	95.98
F6	3578	84	5012	247	4.93	95.07	5013	221	4.41	95.59
F7	3617	81	4891	257	5.25	94.75	4893	198	4.05	95.95
F8	3574	78	4653	255	5.48	94.52	4654	207	4.45	95.55
F9	3652	85	5186	243	4.69	95.31	5187	198	3.82	96.18
F10	3589	82	4912	251	5.11	94.89	4913	203	4.13	95.87
Sum	71,828		91,864	4409	4.77	95.23	91,889	3492	3.78	96.22
